# Resource Recovery Potential From Lignocellulosic Feedstock Upon Lysis With Ionic Liquids

**DOI:** 10.3389/fbioe.2018.00119

**Published:** 2018-09-05

**Authors:** Beatriz Padrino, Marta Lara-Serrano, Silvia Morales-delaRosa, José M. Campos-Martín, José Luis García Fierro, Fernando Martínez, Juan Antonio Melero, Daniel Puyol

**Affiliations:** ^1^Group of Chemical and Environmental Engineering, University Rey Juan Carlos, Mostoles, Spain; ^2^Sustainable Energy and Chemistry Group, Instituto de Catalisis y Petroleoquimica, CSIC, Madrid, Spain

**Keywords:** non-hydric lysis, circular economy, bioenergy, bioproducts, nutrients recovery

## Abstract

Lignocellulosic residues from energy crops offer a high potential to recover bioproducts and biofuels that can be used as raw matter for agriculture activities within a circular economy framework. Anaerobic digestion (AD) is a well-established driver to convert these residues into energy and bioproducts. However, AD of lignocellulosic matter is slow and yields low methane potential, and therefore several pre-treatment methods have been proposed to increase the energy yield of this process. Hereby, we have assessed the pre-treatment of lignocellulosic biomass (barley straw) with the ionic liquid (IL) 1-ethyl-3-methylimidazolium acetate and its effect on the biochemical methane potential (BMP). The BMP of the residue was evaluated at different inoculum to substrate (I/S) ratios and working under meso and thermophilic conditions. Solids destruction upon AD is highly enhanced by the IL-pretreatment. This also resulted in a higher BMP, both in mesophilic as well as thermophilic conditions. At the optimum I/S ratio of 2:1 (dried weight, dw), the BMP of the IL-pre-treated feedstock increased 28 and 80% for 35 days of thermophilic and mesophilic AD, respectively, as compared to the fresh feedstock, achieving values of 364 and 412 LCH_4_/kgTS. We also explored the effect of this pretreatment on the phosphorus recovery potential from the digestate upon release from the AD process. Thermophilic anaerobic digestion of IL-pre-treated biomass provided the highest P recovery potential from lignocellulosic residues (close to 100% of the theoretical P content of the lignocellulosic feedstock). Therefore, the pretreatment of lignocellulosic feedstock with IL before AD is a promising platform to obtain bioenergy and recover P to be regained for the agriculture sector.

## Introduction

The lignocellulosic biomass is abundant and offers a great potential for the production of liquid fuels and chemical products, which have high potential chemical energy to be transformed into biogas by anaerobic digestion (AD) (Sawatdeenarunat et al., 2015). Biogas is another energy source used as car fuel, or for production of heat or electricity in different countries (Ghosh et al., 2000; Sims, 2003). Main lignocellulosic feedstocks are energy crops and agro-forestry residues. The latter offers several advantages due to their low cost, and it does not compete with feed and food production. Moreover, high biomass yields even under low inputs of energy, water, fertilizers, and pesticides, make energy crops ideal for biogas (and bioenergy) production.

However, the main drawback of these feedstocks is their composition, which mainly includes cellulose, hemicellulose and lignin. The interactions of these components create a highly resistant and recalcitrant biomass structure, due to many factors, like lignin content, crystallinity of cellulose, and particle size. These properties considerably decrease the digestibility of the hemicellulose and cellulose present in the lignocellulosic biomass, and in consequence the methane production rate (Mosier et al., 2005; Wilkie, 2008; Agbor et al., 2011; Zheng et al., 2014; Sawatdeenarunat et al., 2015).

In order to avoid this drawback, several pretreatments have been proposed to enhance the digestibility of lignocellulose biomass. There are several studies focused on the chemical deconstructing of lignocellulose, including thermal liquefaction, acid and alkaline hydrolysis, enzymatic hydrolysis, steam explosion, mechanical milling and ammonia fiber expansion, among others (Hendriks and Zeeman, 2009). Each pretreatment has its own effects on the cellulose, hemicellulose and lignin, the three main components of lignocellulosic biomass, and these pre-treatments considerably increasing the treatment costs and the C footprint (Jönsson and Martín, 2016).

A promising pretreatment is the use of ionic liquids (IL) for the deconstructing of the lignocellulosic fibers, which increase the biomass volume and active surface. Ionic liquids are special solvents that can dissolve materials that are otherwise considered insoluble in conventional solvents. They consists of combinations of ions and have special properties such as broader liquid temperature, high thermal stability and negligible vapor pressure, which are the vital properties required in the transformation of lignocellulosic biomass (Galinski et al., 2006; Kassaye et al., 2017). ILs disrupt the non-covalent interactions between lignocellulose components without leading to significant degradation. ILs operation significantly decreases the temperature of the process compared to traditional hydrolytic processes (Morales-delaRosa et al., 2014a). Cellulose regenerated from IL solutions has increased enzymatic convertibility.

The pretreatment with IL favors the digestibility of the lignocellulosic biomass in a same manner than enzymatic hydrolysis, because reduce the crystallinity of cellulose and makes more accessible the cellulose chains for its transformation (Lara-Serrano et al., 2018). Some authors have tried to use IL pretreatment for the anaerobic digestion of lignocellulose (Gao et al., 2013a). These authors propose the pretreatment with a combination of IL+DMSO, which increased the methane production. However, the use of chloride IL and DMSO, can be not adequate because small quantities are toxic for the cell grow (Zhu et al., 2013), then a critical washing step is necessary to avoid this effect that cannot be applied at large scale. To avoid this drawback, the use of non-toxic solvents is mandatory to get a positive effect in the methane production. In addition, the use of these solvents for deconstructing the lignocellulosic material can release other inorganic resources, which has still not been considered and should be also exploited.

The agriculture sector is becoming compromised due to the lack of a cheap and accessible source of inorganic nutrients to sustain the increasing food demand caused by over-population, especially in developing countries (Cordell et al., 2009). Notably, phosphorus represent a key factor for the future sustainability of the agriculture, mainly due to their non-renewable origin (phosphate rock) and the specific location of the main reservoirs in few countries (Cordell et al., 2011). The efforts are being driven toward two main strategies: (a) increasing the efficiency of phosphorus assimilation by crops by minimizing the loses by percolation or runoff (Sharpley, 2016) and (b) recovering the excess of phosphorus dissipated in waste and wastewater sources by intensive recovery technologies based on a biorefinery concept (Tarayre et al., 2016). Main waste sources for P recovery origins from domestic wastewater and livestock waste (mainly manure) (Heilmann et al., 2014). Crop waste has been barely explored as a source of P recovery despite at least 6.4% of P wastage comes from post-harvested crop loses, which represents around 900 k tons P per year worldwide (Cordell et al., 2011). Enabling a complete sustainable agriculture and reducing the pressure on the P extraction from high phosphate rock requires the recovery of this vast P source.

In this work, the pre-treatment of a lignocellulosic feedstock (barley straw) by the non-toxic ionic liquid 1-ethyl-3-methylimidazolium acetate ([C2C1Im][OAc]) and in absence of other solvents was explored as a method to recover energy as biogas and phosphorus as phosphate through anaerobic digestion. Anaerobic digestion was studied at meso and thermophilic conditions, and the effect of the inoculum to substrate (I/S) ratio and the kinetics of the process was analyzed. The experimental data was used for proposing a novel platform to recover resources from lignocellulosic waste.

## Materials and methods

### Materials and chemicals

The ionic liquid [C2C1Im][OAc] (assay ≥95% (HPLC)) was purchased from Sigma Aldrich and used without any further purification or treatment. The sample of lignocellulosic biomass was barley straw. This was provided and characterized by the Unit of Biofuels from the Centre of Energy, Environmental and Technological Research (CIEMAT, Madrid, Spain). It was composed of (% in dw, SD in brackets): extractables, 13.4 (–); aqueous, 11.0 (0.3); organics, 2.4 (0.3); cellulose, 32 (1); hemicellulose, 27.2 (0.4) [composed of xylan, 22.1 (0.5); galactane, 1.30 (0.01); arabinane + mannose, 3.87 (0.03)]; lignin acid-insoluble, 17 (1); lignin acid-soluble, 2.10 (0.03); ash, 3.89 (0.05); acetil groups, 1.72 (0.02). The elemental composition was measured as: C (43.6%), H (5.9%), N (0.8%), S (0.06%), with a moisture of 6.1%.

Mesophilic anaerobic sludge used as inoculum was collected from a full-scale anaerobic digester treating waste sludge located in a domestic wastewater treatment plant (DWWTP) (Móstoles, Spain). Thermophilic inoculum was sourced from the mesophilic inoculum, which was cultivated at 55°C in a lab-scale anaerobic batch reactor for more than 90 day in order to be adapted to thermophilic conditions before the extraction. The batch reactor was fed with activated sludge from the DWWTP aforementioned once per week at a rate of 1 g TS substrate gTS^−1^ inoculum. Once adapted, the thermophilic biomass was collected at the end of every feed round and stored at 4°C before using.

### Ionic liquid pre-treatment of lignocellulosic waste

**Figure 1:**
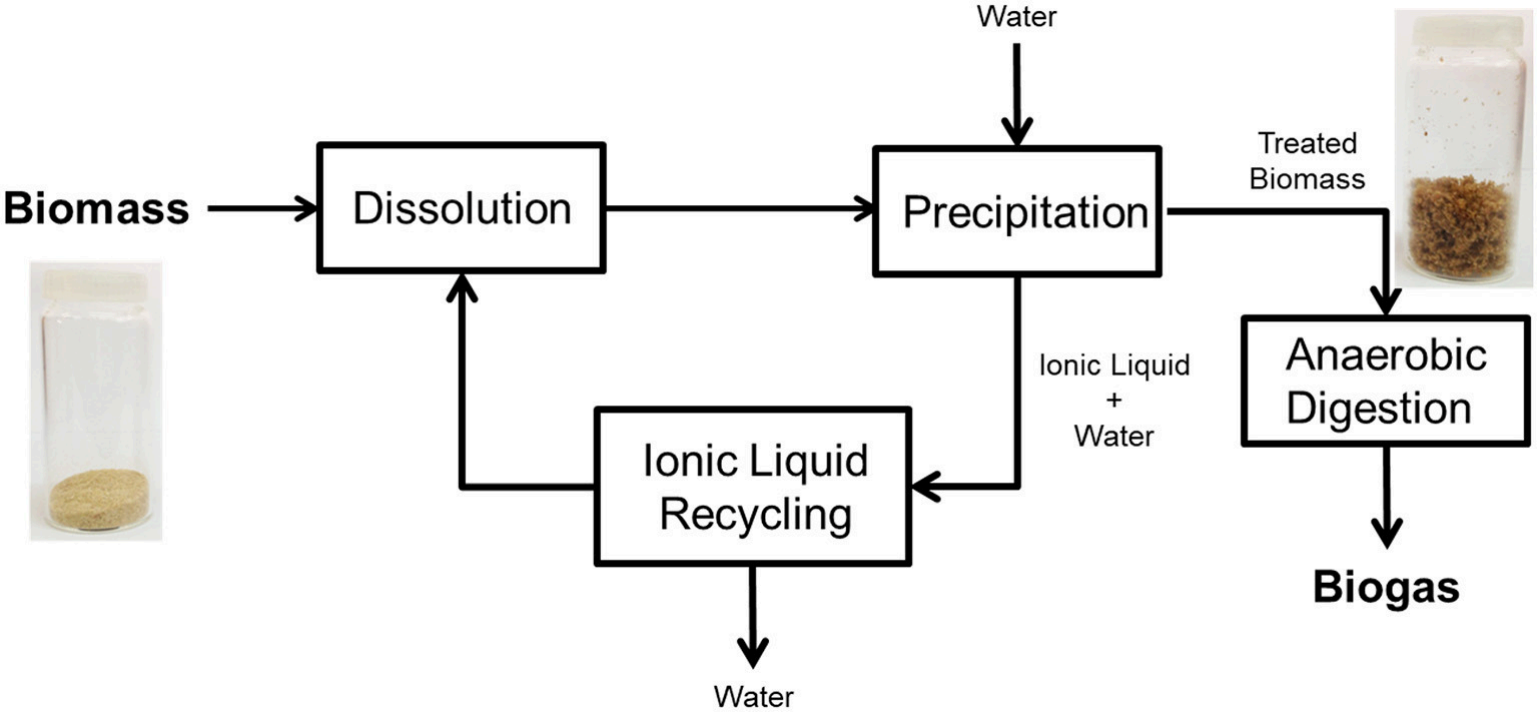
Process scheme for the dissolution of lignocellulosic biomass in ionic liquid and its precipitation via antisolvent strategy to production of biogas by anaerobic digestion.

The treatment of lignocellulosic biomass involved a complete dissolution of barley straw (3 g) in ionic liquid (57 g) at 105°C by using a Mettler-Toledo Easy Max® 102 reactor equipped with mechanical stirring. The complete dissolution was checked by direct visual observation and it happened after 7.5 h. Thereafter, 250 mL of deionized water was added to precipitate the material. The solid obtained was separated by vacuum filtration with a nylon membrane (Morales-delaRosa et al., 2014a, 2015, 2018) and it was washed with deionized water several times in order to eliminate all remaining ionic liquid. Figure 1 shows a schematic view of the process.

### Biochemical methane potential tests

Biochemical methane potential tests (BMPs) were conducted under meso- and thermophilic conditions (37 and 55°C, respectively), according to (Angelidaki et al., 2009). One sixty milliliter flaks serum bottles were inoculated with anaerobic sludge and supplemented with the solid organic substrate to a 1% ds concentration. Bottles were flushed with 99.99% N_2_ gas for 1 min (4 L/min), sealed with a rubber stopper retained with an aluminum crimp seal and stored in temperature-controlled incubators for around 35 days. Tests were mixed by swirling once per day. Two blank tests containing either inoculum and no substrate or substrate and no inoculum were assessed to correct for background methane potential of the added inoculum and to know on the methanability of the substrate with no inoculum, respectively. Inoculum to substrate (I/S) ratios of 2:1, 1:1 and 1:2, expressed as total solids (TS) were tested in the BMP tests. All the tests were conducted in triplicate. All the data are averaged values with 95% confidence intervals.

### Analytical methods

Raw material and solids pre-treated barley straw were characterized at the National Renewable Energy Laboratory (NREL) using the standard methods for determination of structural carbohydrates and lignin in biomass (Sluiter et al., 2010). Moisture content was analyzed by measuring the TS and volatile solids (VS) content according to standard methods (Federation and Association, 2005). ${\text{NH}}_{4}^{+}$ and ${\text{PO}}_{4}^{3-}$ released upon AD experiments were analyzed by Merck Kits (Merck, Kenilworth, NJ, USA) following standard procedures.

Additionally, X-ray diffractions profiles of samples were recorded with an X'Pert Pro PANalytical diffractometer equipped with a CuKα radiation source (λ = 0.15418 nm) and X'Celerator detector based on Real Time multiple Strip (RTMS). The samples were ground and placed on a stainless-steel plate. The diffraction patterns were recorded in steps over a range of Bragg (2θ) between 4° and 90°, at a scanning rate of 0.02° per step and an accumulation time of 50 s. Diffractograms were analyzed with the X'Pert HighScore Plus software.

The scanning electron micrographs of fresh and pre-treated barley straw with IL were taken with a Hitachi S-3000 N instrument. The samples were treated with increasing concentrations of ethanol to fix the structure and to dehydrate the samples. After a critical point drying with a Polaron CPD7501 critical-point drier, the samples were metallized in a Balzers SCD 004 gold-sputter coater; they were sputter-coated with a thin layer of gold.

Biogas production in the BMP tests was measured by pressure increase in the headspace by using a Mariotte oil gas meter (3B Scientific, Spain). Biogas composition was analyzed by a GC/TCD (Agilent Technologies, Santa Clara, USA).

### Kinetic modeling

Kinetic analysis was conducted by using the BMP profiles (expressed as LCH_4_/kgTS added). Maximum biochemical potential (*B*_0_, LCH_4_/kgTS) and hydrolysis constant (*k*_*H*_, 1/d) were calculated by fitting the data to a first order model. The instantaneous biochemical methane potential profiles (*B*) can be defined as the TS fraction of the substrate that is being transformed into methane, according to:

(1) $$\begin{array}{r} B=\frac{S_{CH_{4}}}{S} \end{array}$$

Where S_*CH*4_ is the instantaneous methane production (LCH_4_/L experiment) and *S* is the substrate concentration (kgTS/L experiment). The process will end once all the biodegradable substrate has been transformed into methane, therefore the maximum biochemical methane potential can be defined as the maximum methane production for an infinite time of digestion (*B*_0_).

The model was simplified assuming no biomass growth, and can be summarized as follows:

(2) $$\begin{array}{rcl} \frac{dS}{dt} & = & -k_{H}S \end{array}$$

(3) $$\begin{array}{rcl} \frac{dB}{dt} & = & {-B}_{0}\frac{dS}{dt}=k_{H}B_{0}S \end{array}$$

### Data management

Data from BMP tests are average values with 95% confidence intervals from triplicate measurements.

Calculation of solids destruction (%) was performed according to the Equation (4):

(4) $$\begin{array}{l} Solids\;destruction\;\left(\%\right)\\ =\;\left(1-\frac{\left(TS_{final,\exp}-\left(TS_{initial,\;inoc}-TS_{final,inoc}\right)\right)}{TS_{initial,\;\exp}}\right)*100 \end{array}$$

Where *TS*_*final, exp*_ and *TS*_*initial, exp*_ are the final and initial values of the TS in the BMP of each experiment, whereas *TS*_*final, inoc*_ and *TS*_*initial, inoc*_ are the final and initial values of the TS in the BMP of the inoculum, respectively.

Calculation of P recovery potential was performed by using the following equation:

(5) $$\begin{array}{r} recovery\;potential\;\left(\%\right)=\;\frac{P\;release\;\left(\frac{mgP}{gTS}\right)*0,1\left(\%\right)}{P\;content\;barley\;straw\;\left(\%\right)}*100 \end{array}$$

Where the P content of the barley straw has been calculated from Vassilev et al. (2010) (0.148 % dw).

The mathematical model of the BMP tests was implemented on Aquasim 2.1d. Parameter values and uncertainty analysis were simultaneously estimated, with a 95% CI. Ninety-five percent confidence regions for parameters values were also estimated. Simulation curves were determined by using optimum parameter values. *B* was used as a measured variable, with sum of squared errors as an objective function. All the kinetic analysis was performed as per Segura et al. (2016).

## Results and discussion

### Ionic liquid pretreatment of the barley straw

**Figure 2:**
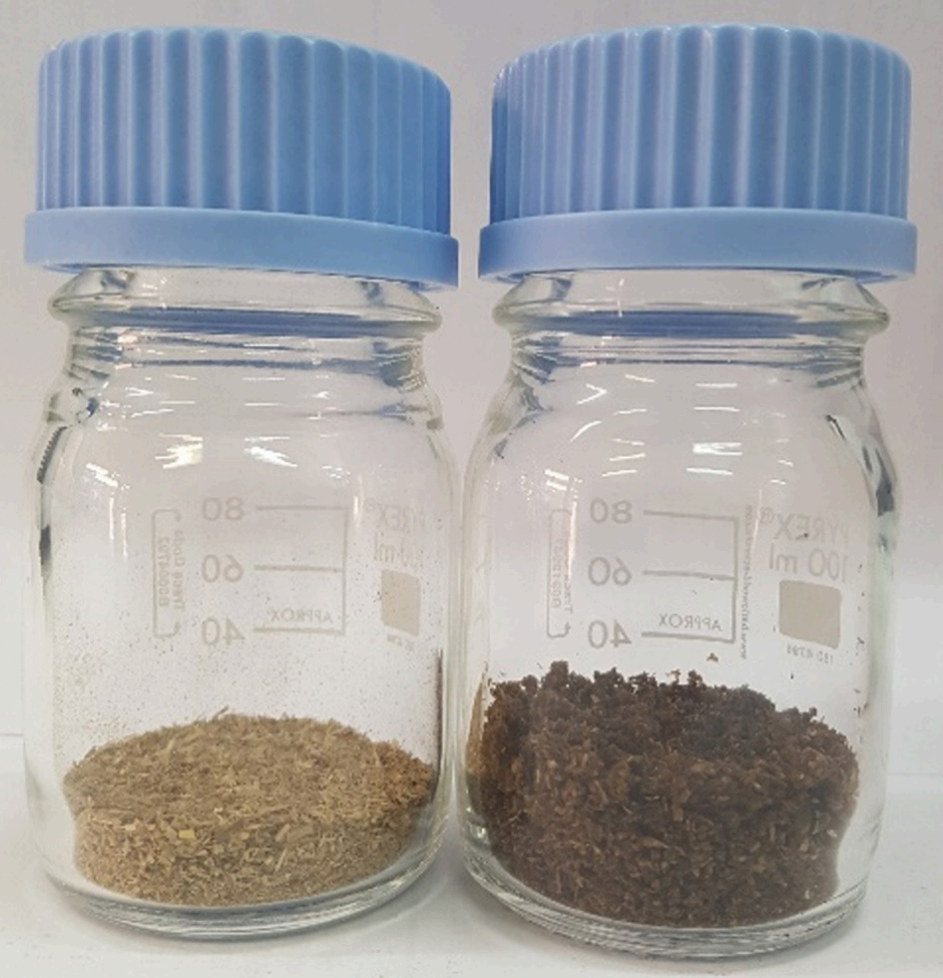
Original **(Left)** and IL-treated biomass **(Right)**.

The dissolution of barley straw in [C2C1Im][OAc], followed by precipitation in water (antisolvent) induced important morphological and textural changes, as already seen in previous works (Morales-delaRosa et al., 2014a, 2015, 2018). The original sample has the typical appearance of sawdust. However, the treated sample is much spongier with a greater volume (Figure 2). There is also a change of color from light to darker brown when the barley straw is treated; it must be considered that the sample is very moist. Moisture of the pre-treated biomass was measured as 89.9% (0.9).

**Figure 3:**
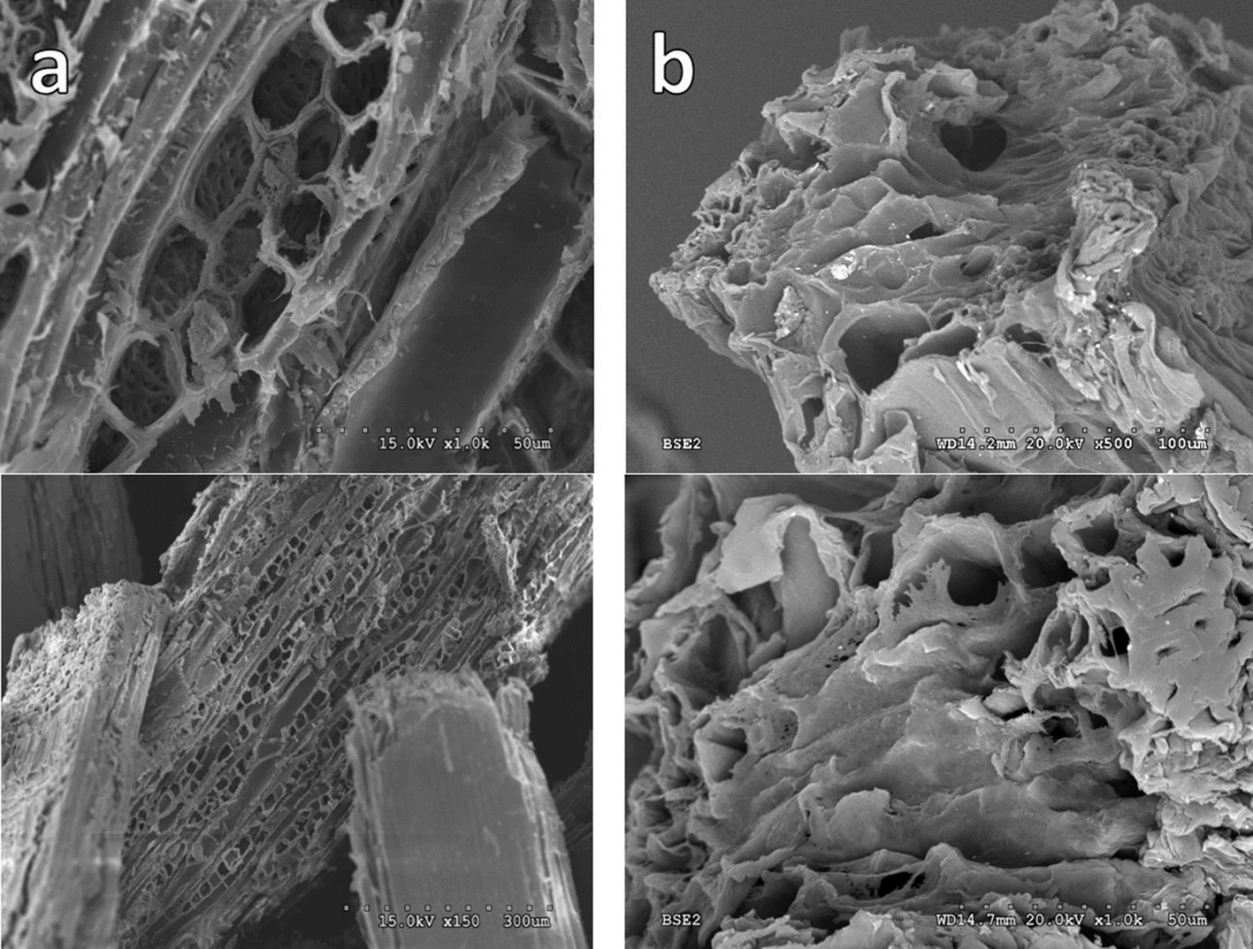
SEM micrographs of Original Barley Straw **(a)** and the barley straw after treatment with IL **(b)**. The pictures below show an enlargement of the same above. The initial structure has been missing but not completely.

SEM images show large differences in the morphology between the original barley straw and the treated with ionic liquid (Figure 3). After 7.5 h, the initial structure of the barley straw has been destroyed. In Figure 3a, the lignocellulosic structure of the barley straw has been seen with the presence of its vascular bundles forming cavities along small pieces. However, that structure is lost in the treated lignocellulosic biomass (Figure 3b).

**Figure 4:**
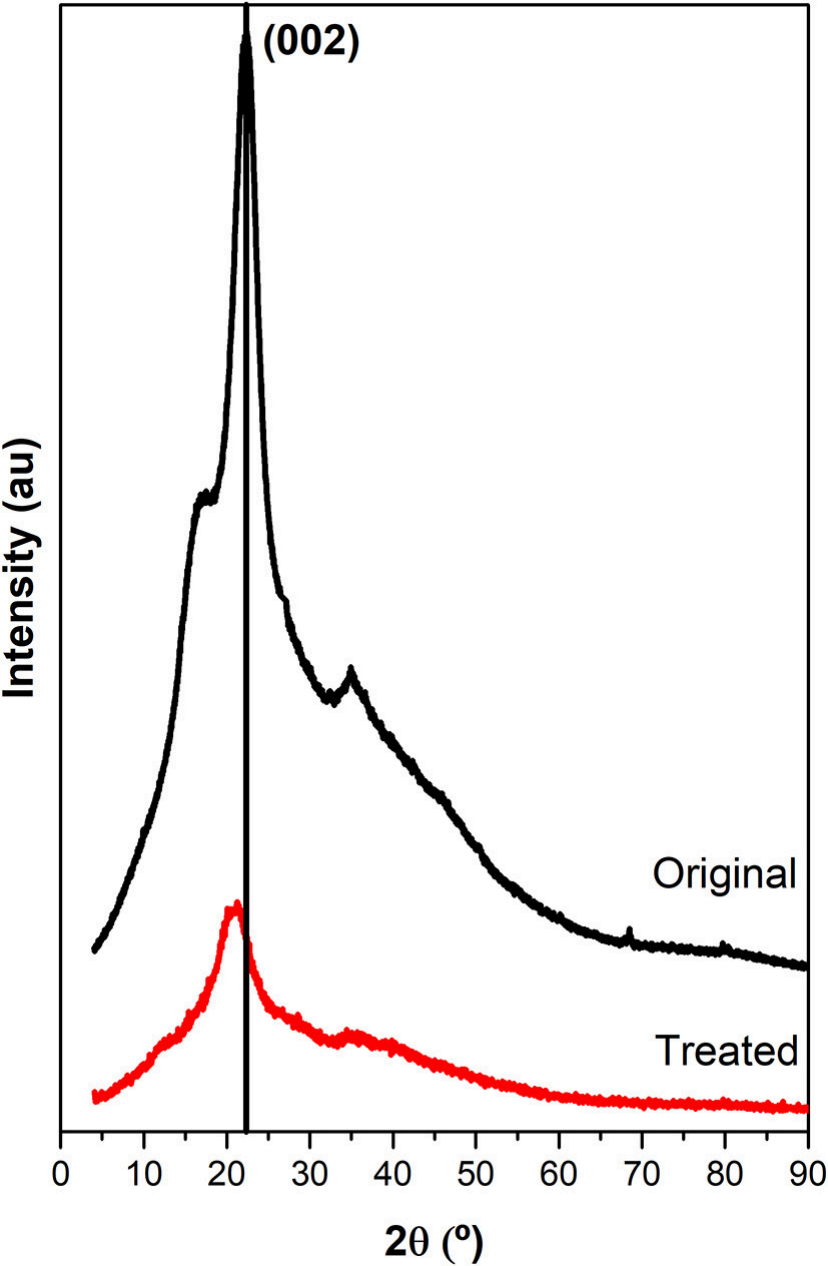
X-ray diffraction for original barley straw and barley straw treated with IL.

X-ray diffraction technique is a powerful tool to analyze the crystalline nature of the materials. Figure 4 shows the comparison of XRD patterns of original barley straw and biomass pre-treated. The diffractogram of untreated barley straw shows typical peaks of the crystalline structure of cellulose, although these peaks are wider and less defined than pure cellulose. This implies the presence of a less orderly or sizes smaller crystal structure. A prominent peak at 23° corresponds with reflection to (200), whereas a peak width between 15° and 17° represents the combination of the two reflections to (1–10) and (110) (Morales-delaRosa et al., 2014b). The intensity of the diffraction peaks losses intensity with the treatment of the sample. Peaks were not observed in the diffractogram of the pre-treated sample, indicating a total disappearance of its crystallinity. This study indicates that the original barley straw has a crystallinity that is lost when treated with ILs (Cheng et al., 2011; Shi et al., 2013).

Therefore, XRD and SEM images showed the loss of crystallinity and a dramatic change in the structure and morphology of the starting material. Thus, at the end of the reconstruction process, the biomass substrate exhibited a much more accessible surface. As the biomass has lost its structure and increased this reactive surface, it was tested as a feedstock for the production of biogas by anaerobic digestion.

### Biochemical methane potential tests

Solids destruction upon the BMP tests (%) was analyzed and compared between fresh and pre-treated biomass. Table 1 shows the values for thermophilic and mesophilic experiments. In general, the solids destruction is much higher (around 2-fold in almost all the cases) in the IL-pre-treated biomass experiments compared to the experiments using fresh biomass. However, there are differences between thermophilic and mesophilic conditions. The I/S ratio does not seem to affect the solids destruction values in thermophilic conditions, indicating that the digestion time is enough to achieve the maximum hydrolysis potential of the biomass even under low I/S ratios (1:2) (around 71–79%). By contrast, the I/S ratio clearly affects the solids destruction in mesophilic conditions. Under I/S ratios of 2:1 and 1:1 the solids destruction is statistically similar in both fresh and IL-pre-treated biomass experiments (yielding 73–85%), whereas an I/S ratio of 1:2 clearly causes a decrease of this value (down to 62%). This is explained by lower hydrolysis rates found under mesophilic conditions (Kim et al., 2003). Therefore, it can be inferred that the pre-treatment of lignocellulosic material greatly enhances the hydrolysis of the biomass, but an excess of substrate causes a drop of the hydrolysis rate in mesophilic conditions. The latter also has important connotations on the release of organics and inorganics and the methane production, as will be furtherly discussed.

**Table 1:** Solid destruction (%) at different I/S ratios in thermophilic and mesophilic conditions upon BMP tests.

**I/S ratio**	**Fresh biomass**	**IL-pre-treated biomass**
**THERMOPHILIC**		
2:1	27 ± 9	74 ± 9
1:1	44 ± 9	71 ± 8
1:2	37 ± 8	79 ± 9
**MESOPHILIC**		
2:1	59 ± 5	73 ± 7
1:1	48 ± 7	85 ± 9
1:2	39 ± 4	62 ± 6

The release of carbon and nutrients upon anaerobic digestion is an important aspect of the potential resource recovery from the digestate. Table 2 shows the release of soluble COD, N as ammonium and P as phosphate, as well as the final pH of the process. Both ammonium and phosphate are the N and P forms that released upon anaerobic digestion, which came mainly from proteins (in the case of N) and from phospholipids and nucleic acids (in the case of P) (Mehta and Batstone, 2013). The low N release associated to the substrate is indicative of the low N content of the biomass (0.8% dw). Indeed, higher ammonium release is observed at increasing I/S ratios, as the N content of the inoculum highly exceeds the lignocellulosic substrate one. In addition, there is no clear relationship between the N release and the pre-treatment or the temperature conditions. P release, however, is comparable or even enhanced at low I/S ratios. This will be furtherly analyzed and detailed below, since this has high impact on P recovery potential.

**Table 2:** Soluble COD, N and P released upon anaerobic digestion in the BMP tests.

	**Control tests**		**BMP tests**		
**Release (mg/L)**	**Substrate**	**Inoculum**	**I/S 2:1**	**I/S 1:1**	**I/S 1:2**
**FRESH BIOMASS**					
**Thermophilic**					
${\text{NH}}_{4}^{+}$-N	5 ± 3	*1, 117*±103	744 ± 26	529 ± 54	325 ± 47
${\text{PO}}_{4}^{3-}$P	4 ± 0.2	41 ± 4	33 ± 1	30 ± 2	33 ± 1
sCOD	294 ± 26	*1, 903*±65	*1, 442*±36	*1, 049*±124	*1, 233*±44
pH	7.4 ± 0.5	7.5 ± 0.4	7.4 ± 0.2	7.8 ± 0.6	7.4 ± 0.4
**Mesophilic**					
${\text{NH}}_{4}^{+}$-N	4 ± 3	1112 ± 91	709 ± 23	664 ± 138	535 ± 178
${\text{PO}}_{4}^{3-}$P	4 ± 0.2	12 ± 1	20 ± 2	23 ± 1	24 ± 2
sCOD	578 ± 34	806 ± 297	712 ± 249	879 ± 181	746 ± 121
pH	7.5 ± 0.8	7.2 ± 0.1	7.7 ± 0.2	7.4 ± 0.2	7.8 ± 0.5
**IL-PRE-TREATED BIOMASS**					
**Thermophilic**					
${\text{NH}}_{4}^{+}$-N	5 ± 0.5	1151 ± 67	783 ± 35	546 ± 81	362 ± 127
${\text{PO}}_{4}^{3-}$P	4 ± 0.3	20 ± 1	33 ± 1	27 ± 2	26 ± 1
sCOD	277 ± 18	*2, 606*±122	*2, 090*±119	*1, 600*±74	*1, 305*±164
pH	8.8 ± 0.4	7.3 ± 0.5	7.9 ± 0.4	8.0 ± 0.2	7.9 ± 0.1
**Mesophilic**					
${\text{NH}}_{4}^{+}$-N	6 ± 4	1243 ± 159	873 ± 54	618 ± 63	324 ± 7
${\text{PO}}_{4}^{3-}$P	2 ± 0.4	20 ± 1	20 ± 2	22 ± 2	25 ± 4
sCOD	319 ± 88	482 ± 72	345 ± 102	235 ± 63	2322 ± 127
pH	8.1 ± 0.3	8.1 ± 0.9	8.2 ± 0.3	8.0 ± 0.3	8.3 ± 0.3

Results of soluble COD (sCOD) evidenced that thermophilic conditions released much more organic compounds than mesophilic conditions. This is usually explained by the solubilization of a proportionally higher fraction of soluble inerts due to higher hydrolysis extent, but also as a result of a proton imbalance due to a fast hydrogen production (in acidogenesis steps) which favors hydrogenotrophic methanogens, which also causes accumulation of volatile fatty acids and alcohols (Franke-Whittle et al., 2014). However, there is no clear trend indicating whether the pre-treatment of the lignocellulosic biomass is favorable or not for avoiding the accumulation of soluble organics, as the trend of sCOD release in both cases is analogous. In contrast, the pre-treatment clearly enhances the biodegradability of the soluble organic matter in mesophilic conditions, as demonstrated with the significantly lower sCOD observed for the I/S ratios of 2:1 and 1:1 in the pre-treated experiments. The higher sCOD in the 1:2 I/S ratio responds to a soluble substrate accumulation due to an excess of substrate, and will be discussed with the methane production data. It can be anticipated that the pH is not a possible cause of inhibition since the final pH in all cases under mesophilic conditions is >7 and therefore acidification does not occur, presumably due to high alkalinity of the process.

**Figure 5:**
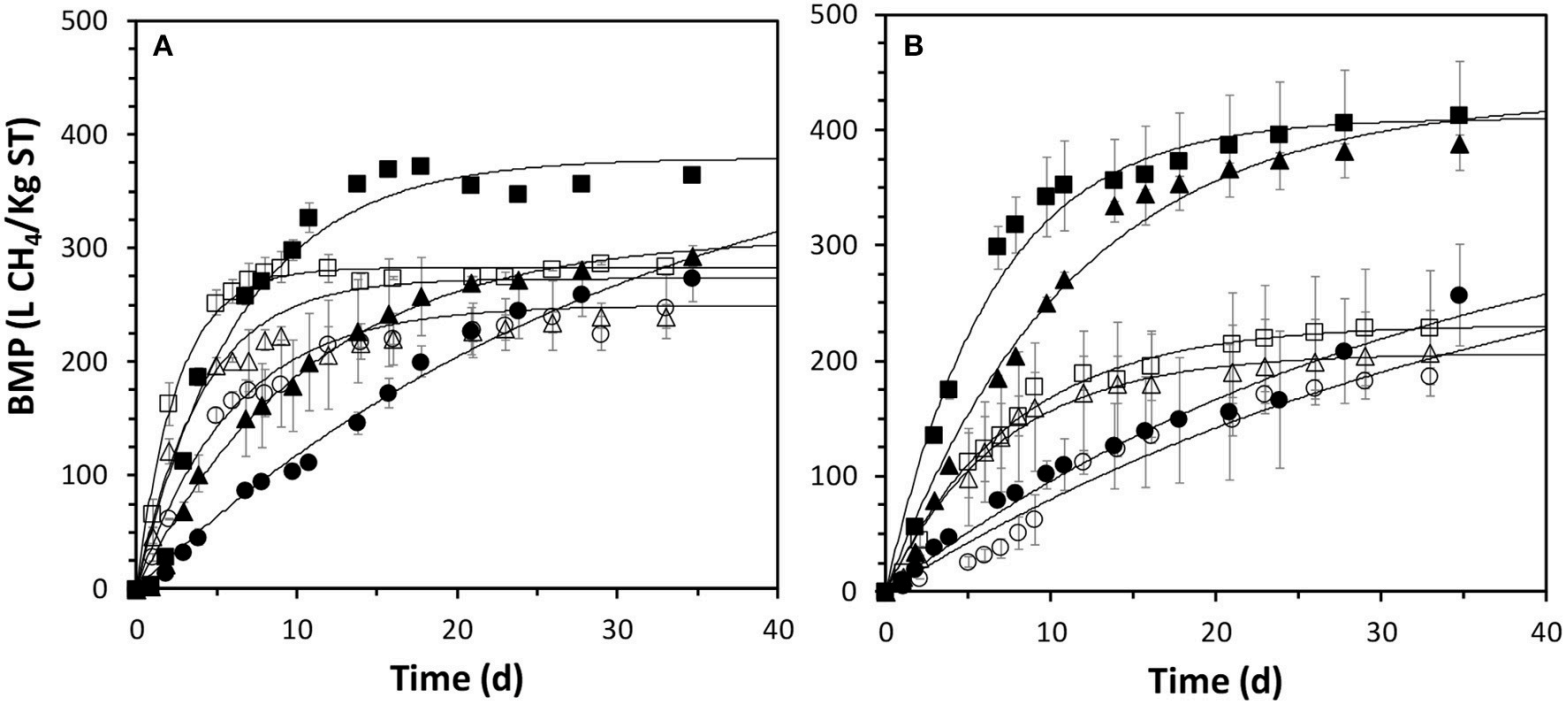
Thermophilic **(A)** and mesophilic **(B)** BMP profiles of fresh (open symbols) and pre-treated (closed symbols) at I/S ratio of 2:1 (squares), 1:1 (triangles) and 1:2 (circles). Error bars are 95% confidence intervals of average values from triplicates. Continuous lines are simulation curves.

Figure 5 depicts the biochemical methane potential profiles. Thermophilic AD of the treated barley straw showed a remarkable methane production for the initial days of the AD at the I/S ratio of 2:1 (Figure 5A). The pre-treated biomass achieved a methane production increase of 28% after 35 day of AD, whereas there were no significantly differences in the BMP at I/S of 1:1 and 1:2. However, the thermophilic process was faster in all cases when the fresh biomass was used. This could be attributed to the fast fermentation of the oligosaccharides due to high temperature, causing accumulation of volatile fatty acids and alcohols that inhibits the methanogenesis (Holliger et al., 2016). This may explain the higher CO_2_ production observed in the pre-treated experiments compared to the fresh experiments (around 2–4% higher in all cases) and the high sCOD at the end of the BMP tests in the pre-treated biomass experiments. All these results evidence an over-activity of fermentative bacteria, which caused high impact on the methane production rate.

In contrast, the IL pre-treatment caused a considerable increase of the BMP of the biomass at mesophilic conditions. BMP increased 77, 108, and 14% for I/S of 2:1, 1:1, and 1:2, respectively (Figure 5B). The higher methane potential of the pre-treated biomass is clearly evidenced. However, a low I/S ratio also caused inhibition problems. In this case of I/S of 1:2, the methanogenesis is inhibited compared to the other I/S ratios. The accumulation of sCOD is an evidence of the inhibited methanogenesis (see Table 2), which also caused a decrease of the solids destruction (see Table 1), therefore affecting also the hydrolytic bacteria. These results as a whole indicate that, in an optimized I/S ratio (or optimized substrate addition rate), the quantity of energy recovered by anaerobic digestion is around 2-fold when the lignocellulosic biomass is pre-treated with 1-ethyl-3-methylimidazolium acetate.

Results obtained in this study are comparably better than most of the few studies found about this subject in the literature (Table 3). We obtained the highest BMP value of any of the values extracted from the literature under mesophilic conditions at an I/S ratio of 2:1 (412 L CH_4_/kgTS), yielding an improvement of 80% with respect to the anaerobic digestion of the untreated feedstock. Most of the studies analyzed used N-Methylmorpholine N-oxide (NMMO) as a solvent to dissolve the cellulose, obtaining improvements of methane production ranging from 14 to 630%, though are normally around 80%, irrespective of the thermal conditions of the BMP tests. However, the digestion time of these studies is considerably longer than the time used in the present study (longer than 40 vs. 35 day of this study), which would be biased the significance of the outcomes extracted (Aslanzadeh et al., 2011; Teghammar et al., 2012; Purwandari et al., 2013; Kabir et al., 2014; Mancini et al., 2018). In addition, NMMO is not considered as an IL system, but a deep eutectic solvent, and therefore its recovery by precipitation with water after the treatment is not possible. Other studies used the solvent system 1-butyl-3-methylimidazolium chloride and dimethylsulfoxide ([C4mim]Cl/DMSO) to dissolve the cellulose, though in the related studies the BMP is analyzed in mesophilic conditions only. The BMP values obtained under these conditions are moderate, and generally much lower than the values obtained in the present study (Gao et al., 2013b). There is very limited information regarding the use of [C2C1Im][OAc] for the improvement of BMP of lignocellulosic waste. A previous work analyzed the BMP of corn stover and switchgrass upon dissolution with [C2C1Im][OAc], obtaining moderate improvement of the BMP with respect to untreated biomass. However, this study used very extended digestion time (120 day), and therefore the effect of the pre-treatment on the kinetics of the AD process was hidden, and only a small increment of methane potential was observed presumably due to the improvement of the biodegradability of the feedstock (Papa et al., 2015). Despite the promising results shown in the literature, there is still strong criticism on the use of ILs for BMP improvement, as is further described below.

**Table 3:** State-of-the-art of best results on the pre-treatment of lignocellulosic waste by organic solvents and ILs and subsequent anaerobic digestion.

Solvent system^*^	**Conditions**	**Lignocellulosic material**	**T (^°^C)**	I/S ratio^**^	***t* (d)**	**BMP (L CH_4_/Kg TS)**	**% Improvement**	**References**
NMMO	120°C, 3 h	Rice straw	37	2:1	40	314	81	Mancini et al., 2016
		Cocoa shell				216	0	
		Hazelnut skin				203	14	
NMMO	120°C, 15 h	Straw fraction of cattle manure	55	NS	52	290	53	Aslanzadeh et al., 2011
		Straw fraction of horse manure			52	333	51	
NMMO	130°C, 15 h	Spruce	55	2:1	42	359	272	Teghammar et al., 2012
		Triticale straw				299	630	
	130°C, 1 h	Rice straw				185	583	
NMMO	120°C, 3 h	Oil palm empty fruit bunch	55	2.7:1	50	347	48	Purwandari et al., 2013
NMMO	90°C, 7 h	Barley straw	55	NS	45	189	92	Kabir et al., 2014
	90°C, 30 h	Forest residues	55		45	128	88	
[C4mim]Cl/DMSO	120°C, 2 h	Water hyacinth	35	NS	30	158.1	98	Gao et al., 2013a
[C4mim]Cl/DMSO	120°C, 2 h	Water hyacinth	35	NS	30	80	64	Gao et al., 2013b
		Rice straw				129	70	
		Mango leaves				68	65	
		Spruce				117	66	
[C2C1Im][OAc]	100°C, 3 h	Corn stover	37	2.4:1	120	322	19	Papa et al., 2015
		Switchgrass				290	14	
	90°C, 30 h	Forest residues	55		45	128	88	
[C2C1Im][OAc]	105°C, 7 h	Barley straw	37	2:1	35	412	80	This work
			37	1:1		388	88	
			37	1:2		257	38	
			55	2:1		364	28	
			55	1:1		293	22	
			55	1:2		274	10	

* *NMMO, N-Methylmorpholine N-oxide*,

** *Inoculum to Substrate ratio in dw basis*.

### Drawbacks and potentials of lignocellulosic IL pre-treatment

Main drawbacks of the use of ILs are related with the limited knowledge on the effect of the dissolution process on the AD performance and a general lack of optimization procedures. However, these drawbacks can be overcome through dedicated optimization and process tuning.

The lack of nutrients can hinder the AD process of IL-pre-treated lignocellulosic feedstock in a long-time frame due to the elemental composition of this feedstock, which mainly lacks a N source to sustain bacterial growth. As an alternative to chemical pre-treatments, co-digestion of this biomass with N-rich waste (as manure) would provide N and trace elements to the AD process (Mao et al., 2017). However, the use of highly alkaline co-substrates can derive in problems due to high pH values, and can maximize the toxic effect of free ammonia, especially at high ${\text{NH}}_{4}^{+}$ concentrations (Hansen et al., 1998). In contrast, as shown recently, direct chemical pre-treatment of lignocellulosic biomass seems to be more effective than addition of extra trace elements (Mancini et al., 2018). This is due to the low nutrients requirements of methanogens in comparison with the requirements of hydrolytic and fermentative bacteria. The chemical pre-treatments destroy the structure of lignocellulosic material, solubilize the biomass and reduce the need for high nutrients-dependent hydrolytic and fermentative processes. This in turn decreases the number of steps needed to transform the organic carbon into methane by methanogens, and therefore increases the methane potential of the lignocellulosic feedstock.

Another potential drawback is the need for cleaning the lignocellulosic feedstock after the pre-treatment to avoid residual ILs in the structure, as there are clear evidences indicating that these compounds are highly inhibitory to methanogenic consortia (Li and Xu, 2017; Li et al., 2017), even to cellulose hydrolyzers (Zhu et al., 2013). However, recent advances have found that it is possible to enrich a consortium that is resistant to the toxicity of the ILs on the cellulose activity (Tantayotai et al., 2016), strongly indicating that an AD consortia can be adapted to IL-bearing lignocellulosic waste after long periods of continuous adaptation. For example, UASB-type reactors can be used, where the hydrolytic bacteria are commonly found in the external layers of the anaerobic granules, and can act as a protective barrier against the toxicity of residual ILs to more sensitive methanogens.

In any case, all these drawbacks are related to an obvious lack of optimization procedures, as this technology is still in its infancy. More studies are needed for knowing the real effect of the IL pre-treatment on the AD of lignocellulosic materials, as there is very limited information regarding continuous treatment of these materials by AD processes. A recent study showed the continuous treatment of raw and pre-treated with NMMO lignocellulosic waste textiles by a two-stage AnSBR-UASB anaerobic process, where the pre-treatment achieved almost double methane production compared to the process using raw waste (400 vs. 200 L CH4/kg VS d) (Jeihanipour et al., 2013). This reflects the potential of this promising technology, though field demonstration is still needed.

### Kinetics of the anaerobic digestion process

**Figure 6:**
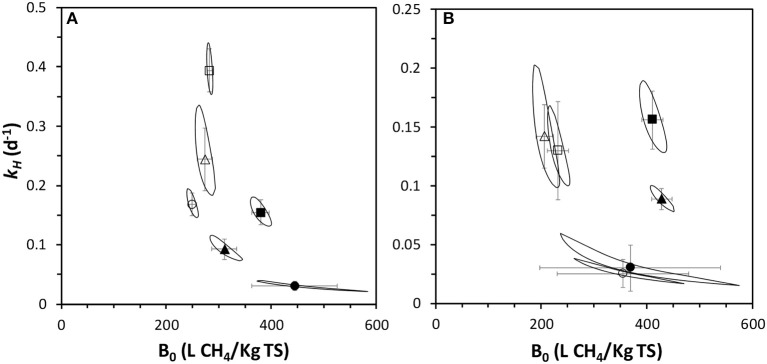
Kinetic parameters of thermophilic **(A)** and mesophilic **(B)** BMP profiles of fresh (open symbols) and pre-treated (closed symbols) at I/S ratio of 2:1 (squares), 1:1 (triangles) and 1:2 (circles). Error bars are 95% confidence intervals from parameter estimation. Continuous lines are 95% confidence regions for *k*_*H*_/*B*_0_ intercept.

A kinetic analysis was performed on the BMP tests data. The parameters estimation for a first order model was carried out and the values and 95% confidence intervals are depicted in Figure 6, along with the 95% confidence surfaces of the *k*_*H*_/*B*_0_ intercept. The first order model accurately predicts the BMP profiles. Simulation curves shown in Figure 5 indicates that the goodness of fitting is acceptable (R^2^ higher than 0.95 in all cases). In addition, relatively low 95% confidence intervals for the parameters values are estimated. Therefore, this simplified model and the parameters values can be used for the analysis and comparison of the AD performance in the different BMP tested, as has been previously suggested (Angelidaki et al., 2009).

The kinetic analysis confirmed that the IL treatment only increased slightly the methane potential in thermophilic conditions (Figure 6A). A *B*_0_ value of 379 ± 16 LCH_4_/kgTS for a I/S of 2:1 was obtained after IL pre-treatment, which is higher than other values obtained for energy crops at thermophilic conditions (Meng et al., 2016).

However, as previously mentioned, the hydrolysis rate considerable decreased, presumably due to the solubilisation of polysaccharides that promoted the growth of thermophilic fermentative bacteria, decreasing the pH. Indeed, the soluble COD at the end of the experiments resulted from the feedstock was much higher with the IL-treated feedstocks: 369 and 296 vs. 185 and 98 mgCOD L^−1^ at I/S of 2:1 and 1:1 for pre-treated and fresh biomass, respectively. Relatively low pH (< 7) was generally observed after the thermophilic treatments, being lower than 6 (5.8) in the case of the I/S ratio of 1:2, which concurs with the lowest *B*_0_ values calculated at this I/S ratio, regardless of the pre-treatment.

Kinetic parameters and statistical analysis of mesophilic BMPs are depicted in Figure 6B. This analysis corroborates that the IL pre-treatment increased considerably the methane potential of this feedstock. *B*_0_ values of 410 ± 20 and 418 ± 19 LCH_4_/kgTS at I/S of 2:1 and 1:1 after IL pre-treatment contrasted positively with those values obtained with the fresh sludge, 282 ± 17 and 240 ± 40 LCH_4_/KgTS, respectively.

In addition, there were no statistical differences in the *k*_*H*_ at I/S of 2:1 and 1:1, remaining around 0.10–0.15 1/d, which concurs with typical values obtained in energy crops (Meng et al., 2016). The I/S of 1:2 considerably decreased the *k*_*H*_ values, which may be due to biomass transfer problems due to bad mixing or inhibition by organic acids accumulation, as has been claimed previously in BMP standardization procedures (Holliger et al., 2016).

### Nutrients recovery potential

**Figure 7:**
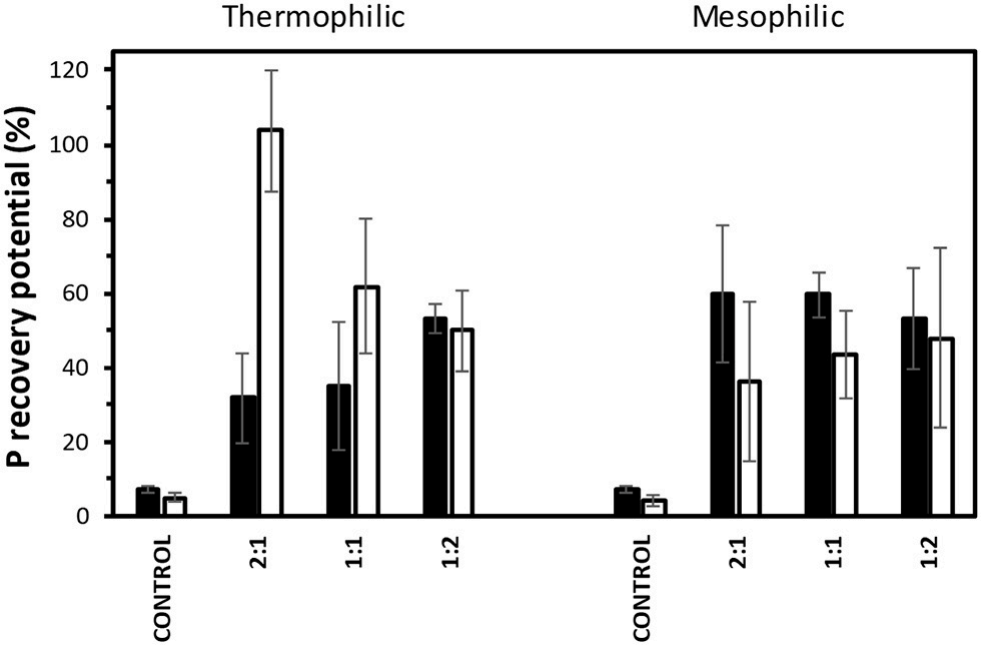
Phosphorus recovery potential upon anaerobic digestion of fresh (black bars) and IL-pre-treated (white bars) barley straw in thermophilic and mesophilic conditions at different I/S ratios. Error bars are 95% confidence intervals.

As commented before, the release of nutrients upon the AD process is an opportunity to regain these compounds to be used furtherly in industrial or agroforestry applications, especially N and P. N recovery seems to be unreliable due to the low N content of the biomass. P recovery, however, sounds promising and can be a driver to an agroforestry-based biorefinery process development. Figure 7 shows the P recovery potential from the digestate after AD between the fresh and pre-treated biomass samples. In general, there are clear differences on the P recovery potential in function of the thermal conditions of the AD treatment and their relationship with the I/S ratio. However, these differences are less significant for the mesophilic conditions in both fresh and IL-pre-treated biomass samples regarding the I/S ratio. The P recovery potential of the fresh biomass upon AD remains around 50–55%, whereas this value slightly reduced down to around 35–40% in the case of the IL-pre-treated biomass, irrespective to the I/S ratios. In thermophilic conditions, both the I/S ratio and the pre-treatment clearly modify the P recovery potential. Low I/S ratios seem to enhance the P release for the fresh biomass, since the P recovery potential is increased from around 30% at a I/S ratio of 2:1 to around 50% at a I/S ratio of 1:2. In contrast, and surprisingly, the pre-treated biomass displayed the opposite behavior. At I/S ratio of 2:1 the P recovery potential is very high (close to the theoretical 100%), whereas these values decreased down to around 50% for an I/S ratio of 1:2. In summary, it can be concluded that the best conditions for maximizing the P recovery potential are pretreating the biomass with ILs and applying thermophilic digestion at high I/S ratios.

### Resources recovery from barley straw

The classical concept about AD as a driver to resource recovery is changing rapidly. It is well stablished that the AD process is a mature and reliable way of extracting and leveraging the chemical energy contained in the residual waste, irrespective to its origin (Puyol et al., 2017). AD has been exploited as an alternative and sustainable way of obtaining energy from renewable sources. Energy crops are among the most typical biomass sources to be used for this purpose, though this has ethical connotations in which second-generation biofuels are direct competitors of food-based agriculture (Tenenbaum, 2008). However, the AD concept has been upgraded to deal with the production of energy from biomass of residual origin, thereby avoiding the competition with food production. The direct production of energy from barley straw is a direct example of this concept.

Nevertheless, novel concepts for resource recovery are dealing with the paradigm of the circular economy, where the residues from the productive system must be completely re-introduced in the market as raw matter (Puyol et al., 2017). In the agroforestry industry, the energy recovery is a necessary resource but is low-cost, and it cannot solve the problem of fertilizers depletion. In this sense, it is necessary to re-conceptualize the use of the AD for agroforestry waste treatment by proposing new ways of recovering high value-added resources from this waste, notably sustainable fertilizers (Batstone and Virdis, 2014).

**Figure 8:**
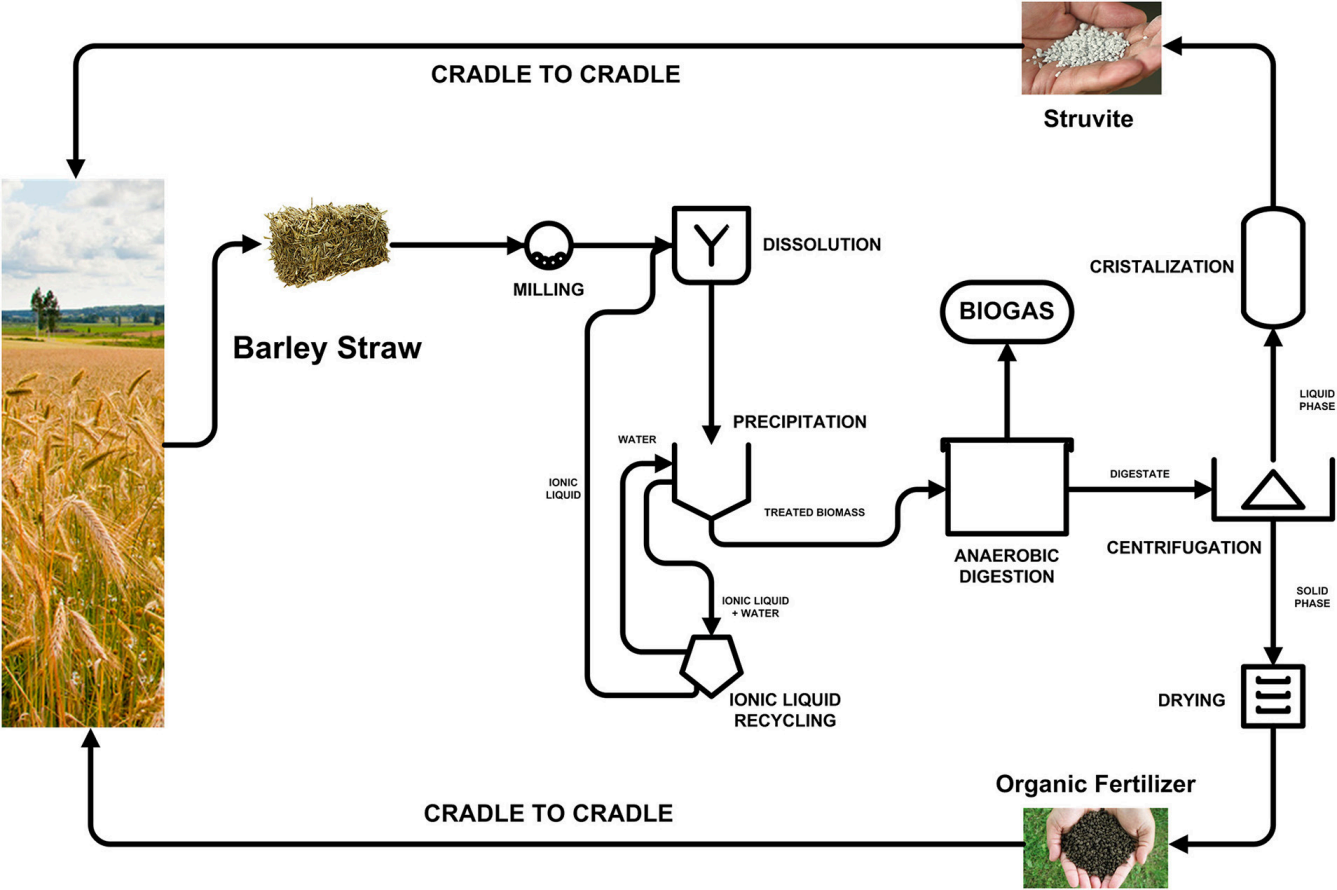
Schematic process for resource recovery from barley straw by ionic liquid dissolution and anaerobic digestion.

Based on the results obtained in this work, we propose the use of the AD process as a core part of a novel strategy of energy and P recovery from the barley straw, in particular. This biorefinery concept is summarized in Figure 8. The barley straw is collected from the farm activities and milled to reduce and homogenize its size. Thereby, IL-based pre-treatment is applied to the straw to deconstruct lignocellulosic matter. The addition of water leads to the precipitation of the treated biomass, which is furtherly separated from the IL. The IL is then recovered and reused in a circular way. The treated biomass is anaerobically digested in thermal conditions at high sludge retention times to have a high I/S ratio (SRT above 35 day). Thereby, a value close to 400 L CH_4_/kg TS has been achieved and, most interestingly, the P contained in the biomass was almost completely released and mobilized into the digestate (as has been demonstrated in this work). Commercial technologies can be applied to recover the soluble P as struvite from the liquid fraction of the digestate. The solid fraction of the digestate can be directly converted into organic fertilizer by air-drying. Both resources and the water excess can be re-introduced into the market and serve as fertilizers for sustainable and bio-economical agriculture, which close the circle.

The key part of the concept, due to reactants consumption and energy needs, is the IL pre-treatment. As the technology is still in its infancy, with an optimistic technological readiness level of 3, the economics of the process can only be derived from theoretical analyses or lab-scales mass and energy balances approximations. As pointed out previously, the aim is to reduce the costs below $1/kg IL, with >97% of IL recovery and >90% of waste heat recovery (Baral and Shah, 2016). These are very optimistic considerations, and the technology requires further development. However, techno-economic assessments still do not include the P recovery, and decrease of C footprint due to mild conditions (Segura et al., 2016). There are also other potential IL alternatives that may reduce considerably the IL costs. George et al. (2015) used the low cost IL triethylammonium hydrogen sulfate as an alternative to the commercial 1-ethyl-3-methylimidazolium acetate, achieving around 75% efficiency of the commercial IL, demonstrating that some ILs can compete with the cheapest pretreatment chemicals, such as ammonia, in terms of effectiveness and process cost. A further scale-up of the technology using this low-cost IL has been tested in simulation, and results based on a continuous lab-scale process demonstrated that is possible to reduce the IL costs below $1/kg IL with a >97% IL recovery. However, heat recovery is not contemplated in that simulation (Brandt-Talbot et al., 2017). In the concept proposed here the AD process under thermophilic conditions is a high heat-demanding process. The optimization of the heat recovery should be driven toward making the most of the heat excess during the AD process.

## Conclusions

The effect of pre-treatment of lignocellulosic feedstock (barley straw) by the IL [C2C1Im][OAc] on the bioenergy (biogas) and P recovery potentials has been analyzed through dedicated BMP tests and mass balances analysis. Main outcomes of this work are listed below:

- Deconstruction of lignocellulosic feedstock by IL causes an increase in the BMP, achieving values of 412 and 364 L CH_4_/kg TS for a I/S ratio of 2:1 in mesophilic and thermophilic conditions, respectively.
- High solubilization of the feedstock by IL-pretreatment produces inhibition of the methanogenesis rate due to excess of the acidogenesis process, especially at thermophilic conditions. This can be controlled by applying an adequate I/S ratio.
- The IL-pretreatment increases considerably the solids destruction and also enhances the P recovery potential, especially upon the thermophilic AD, where P solubilization is close to the theoretical 100% P balance.

## Author contributions

DP designed the experiments and redacted the manuscript. JC-M designed the experiments and reviewed the manuscript. BP, ML-S, and SM performed the experimental analyses. JF, FM, and JM critically reviewed the manuscript. DP and JC-M are both corresponding authors.

### Conflict of interest statement

The authors declare that the research was conducted in the absence of any commercial or financial relationships that could be construed as a potential conflict of interest.
